# Two cases of contact athletes with anterior cruciate ligament injuries who returned to competition early after conservative treatment with PRP therapy

**DOI:** 10.1016/j.ijscr.2022.107268

**Published:** 2022-06-01

**Authors:** Shinnosuke Hada, Muneaki Ishijima, Hiroyuki Omiya, Yoshimasa Tomita, Masao Hada

**Affiliations:** a Dept. of Orthopaedic Surgery, Tokyo Rosai Hospital, Tokyo, Japan; b Dept. of Orthopaedic Surgery, Juntendo University School of Medicine, Tokyo, Japan; c Hada Medical Clinic, Tokyo, Japan; d Dept. of Rehabilitation, Tokyo Rosai Hospital, Tokyo, Japan

**Keywords:** Platelet-rich plasma, Autologous biotherapy, Anterior cruciate ligament, Conservative treatment, Return to play

## Abstract

**Introduction:**

Anterior cruciate ligament (ACL) injuries in high-impact contact sports athletes are often treated through surgery. Recently, conservative treatment of ACL injuries in athletes using autologous biotherapy such as platelet-rich plasma (PRP) has been reported. We report two cases of aggressive conservative treatment with PRP therapy for contact athletes.

**Presentation of case:**

Case 1. A 23-year-old male rugby player with a preinjury Tegner score of 9 underwent three PRP treatments following an ACL injury and returned to preinjury levels of play 4 months postinjury. The pivot shift test had improved to grade 0 at the last follow-up. Case 2. A 34-year-old male mixed martial arts fighter with a preinjury Tegner score of 9 underwent five PRP treatments following an ACL injury and returned to his preinjury play level 3.5 months postinjury. Both contact athletes returned to competition early and continued to compete without losing stability at the final follow-up. Moreover, their functional score exceeded the median score for athletes after ACL reconstruction, suggesting satisfactory performance.

**Discussion:**

Antiinflammatory cytokines and growth factors in PRP may promote graft maturation and alter the natural history of remodeling, resulting in earlier and firmer graft maturation, improving both the duration and quality of the healing process.

**Conclusion:**

Two high-impact sports athletes with ACL injuries were treated with aggressive conservative therapy using PRP and returned early to their preinjury play level.

## Highlights

• Conservative treatment of ACL injuries with platelet-rich plasma
• Two cases of aggressive conservative treatment of contact athletes with PRP therapy
• They returned to the same preinjury level as early as 4 and 3.5 months.
• Their functional score exceeded the median score of ACL reconstructed athletes.
• The growth factors in PRP may promote graft maturation of ruptured ACL.

## 1 Introduction

The two treatment options for anterior cruciate ligament (ACL) injuries are surgical and conservative, depending on the patient's activity and background. Conservative and surgical treatments do not differ markedly in the long-term follow-up of the progression of knee osteoarthritis [1], [2]. Conversely, in a study of active patients, the recovery of the Tegner activity score was better in the surgical group than in the conservative group [3], [4], and the risk of residual instability and meniscal tears with conservative follow-up of ACL injuries was nearly 30 % [4], [5], [6]. High-activity young adults are usually recommended surgery, and conservative treatment is rarely preferred, particularly in high-impact sports such as rugby and martial arts, which involve cutting movements and contact forces and high-level athletes. Typically, conservative treatment is recommended for recreational-level patients, low-impact sports, and middle-aged and older adults [3]. Nevertheless, surgical treatment has a long rehabilitation period before returning to competition, and, in some cases, depending on the athletes' social background, they choose conservative treatment. Although orthotics and physiotherapy has been the mainstay in conservative treatment, results based on the application of autologous biotherapy have gradually started appearing. Specifically, platelet-rich plasma (PRP), prepared by centrifuging autologous blood and concentrating platelets to a high degree [7], has garnered considerable attention and is reportedly applied to the conservative treatment of ACL injuries utilizing the growth factors and antiinflammatory cytokines in platelets to repair damaged tissues [8], [9]. Nonetheless, the indications and limitations of PRP remain unclear, and additional cases need to be accumulated. Primarily, it is crucial to determine whether it is feasible for high-impact sports athletes who are at high risk of reinjury to return to play safely and quickly. Here, we report two cases in which aggressive conservative therapy using PRP was applied to ACL injuries in high-impact sports athletes, who returned to the same level of preinjury play. In order to establish effective non-surgical treatment options for ACL injuries in the future, this article examines the effectiveness of PRP treatment in contact athletes. This report is a retrospective study, approved by the Ethics Committee (03-20) and has been reported in line with the SCARE criteria, and written informed consent was obtained from the patient for the use and publication of data for academic purposes. A copy of the written consent is available for review by the Editor-in-Chief of this journal on request [10].

## 2 Treatment process

PRP was prepared using MyCells® (Kaylight Ltd., Ramat-Hasharon, Israel) and GPSIII® (Zimmer Biomet Holdings, Inc. Indiana, USA). Peripheral blood (20 mL–52 mL) was collected and centrifuged at room temperature to extract nearly 3–6 mL of PRP. Depending on the patient's condition, PRP was injected into the joint and around the joints as often as necessary. Rehabilitation was started on the day of consultation without any restriction of load or range of motion, and a simple semirigid knee brace was available on the market. The intensity of rehabilitation was increased according to physical findings, muscle recovery, and the player's fear and anxiety, the Knee injury and Osteoarthritis Outcome Score (KOOS) [11] was used as a reference. The KOOS is a 42-itemself-reported, joint-specific questionnaire which comprises five subscales: pain (P), symptoms (S), activities of daily living (A), sport and recreation (SP) and quality of life (Q). Each item is scored from 0 (no problems) to 4 (extreme problems). For each subscale, the score was normalized to a 0–100 scale with higher scores indicating better status. The timing of return to play was determined by the Lachman test negative, improvement of the pivot shift test to grade 0 or 1, and all subscales of KOOS exceeded the median score (pain:73.5, Symptoms:62.0, ADL:72.5, unction in Sport and Recreation:52.0, QoL:54.5) in competitive athletes after ACL reconstruction (7.6 months) [11]. The same doctor diagnosed and treated the two cases.

## 3 Presentation of Case 1

**Fig. 1:**
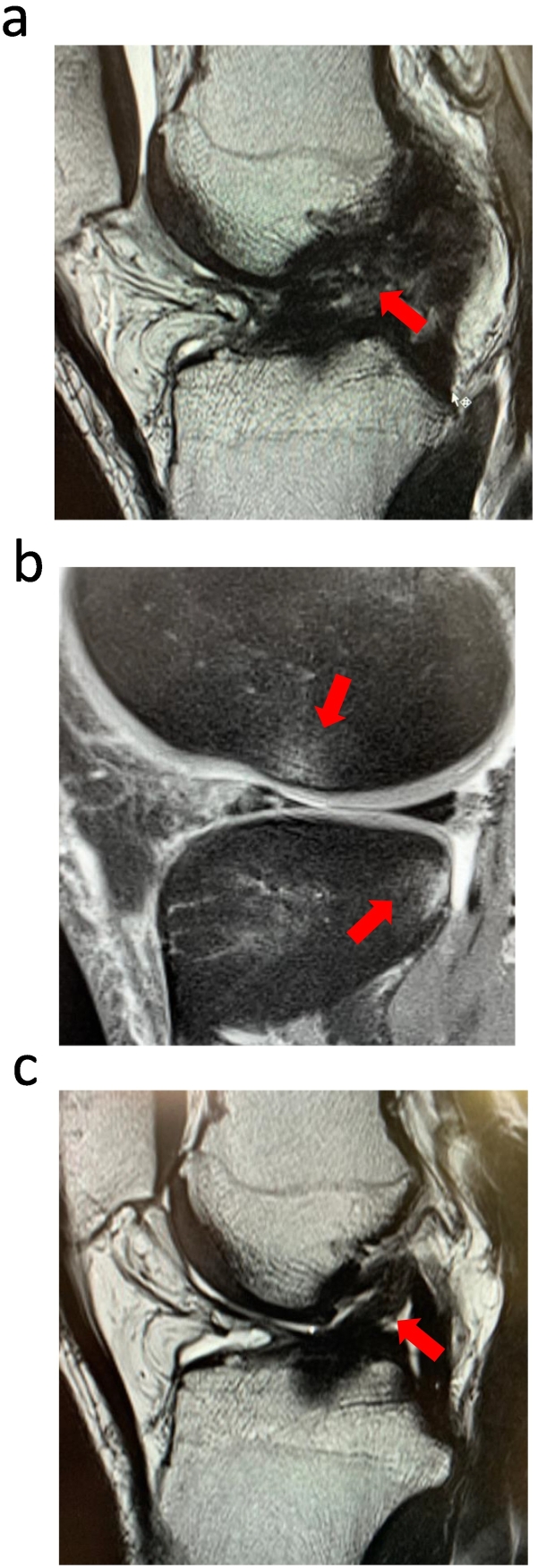
a ACL at the time of injury. b Bone bruise. c ACL at 1 year follow-up.

A 23-year-old male rugby player with a preinjury Tegner score [12] of 9 was injured in the right knee by an opponent's tackle and was seen as an outpatient 1 week later. The Lachman test was positive, the pivot shift test was grade 2, and arthrocentesis revealed 60 cm^3^ of bloody drainage. MRI revealed a median rupture of the ACL and a bone bruise of the lateral femoral condyle (Fig. 1a and b). The patient was offered surgical reconstruction and suggested that surgery would be inevitable owing to sports demands and level. Upon discussing the pros and cons of treatment options, the athlete chose the nonoperative treatment route, reevaluating progress at regular intervals and acknowledging that an episode of instability at any time in the future might lead to surgical reconstruction and possibly further injury to the meniscus and/or cartilage. He had experience with PRP treatment for other injuries and wanted to use it for this injury. He underwent three PRP treatments at 2, 3, and 5 weeks postinjury. He started jogging 9 weeks postinjury, returned to all exercises with a grade 1 pivot shift test at 14 weeks, and returned to the game with a KOOS of 98.8 (P: 100.0, S: 96.4, A: 100.0, SP: 100.0, Q: 93.8) at 18 weeks, recovering to a Tegner score of 9, the preinjury level (Fig. 3a). At 1-year follow-up, the regenerated ACL was clearly visible on MRI (Fig. 1c), and the pivot shift test was grade 0. At the final 3-year follow-up, the patient was still competing without reinjury or compromising.

## 4 Presentation of Case 2

**Fig. 2:**
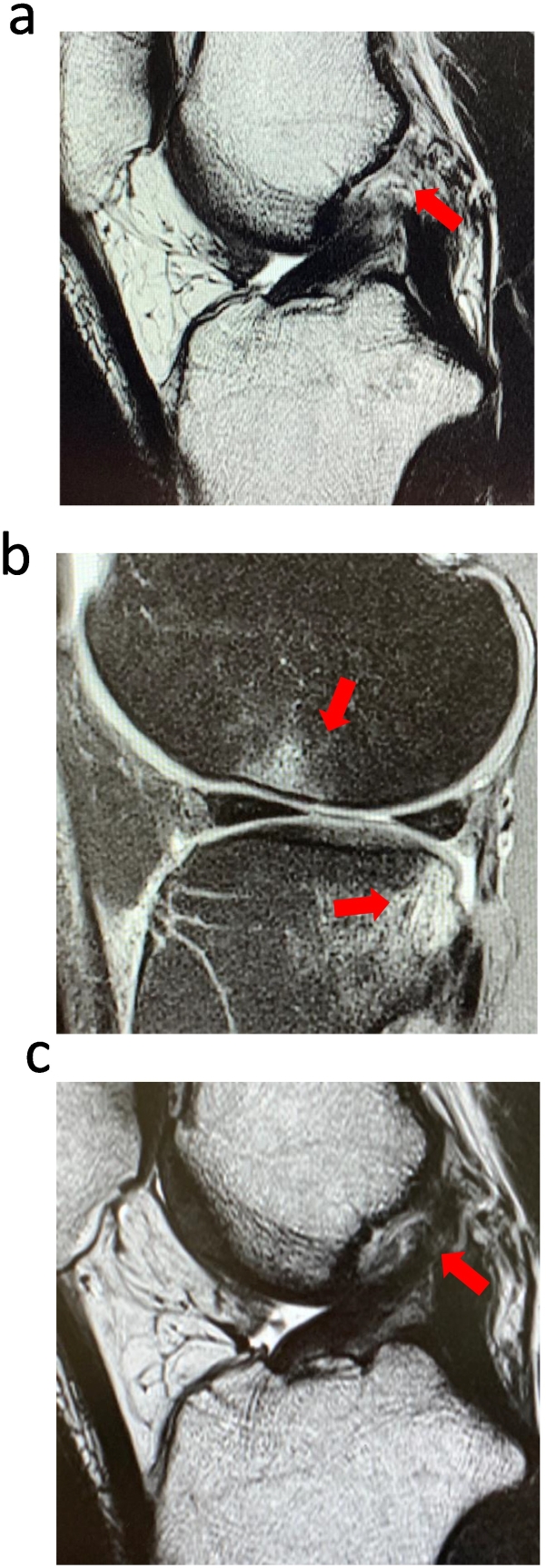
a ACL at the time of injury. b Bone bruise. c ACL at 4 months follow-up.

**Fig. 3:**
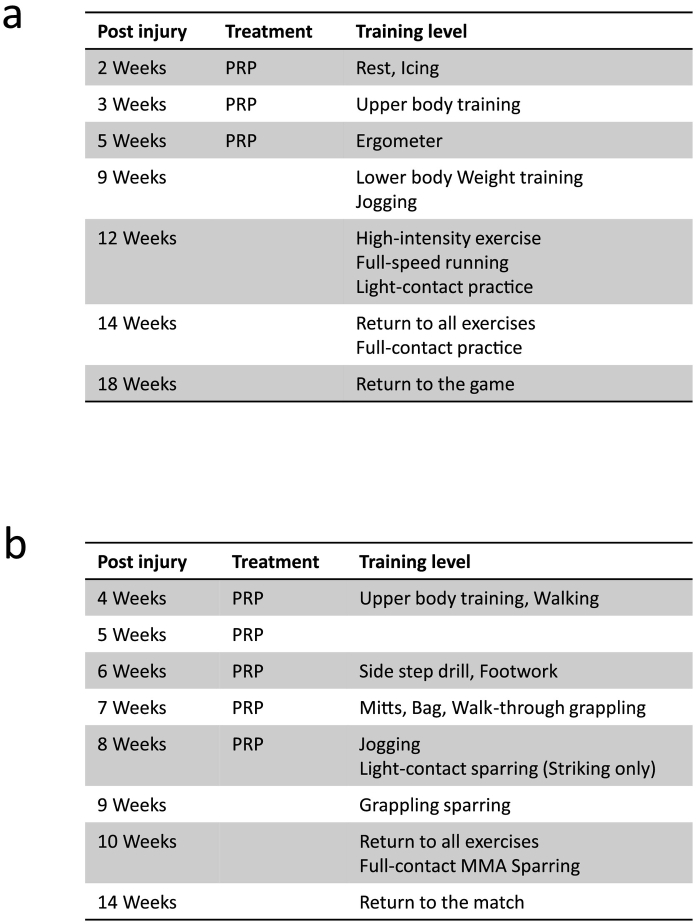
a Return schedule of Case 1. b Return schedule of Case 2.

A 34-year-old male mixed martial arts fighter with a preinjury Tegner score of 9 was injured when thrown by his opponent in a fight. His previous doctor diagnosed an ACL injury and recommended surgery, but for reasons similar to Case 1, he insisted on conservative treatment with PRP. 4 weeks postinjury, he had an outpatient visit to our hospital. The Lachman test was positive, and the pivot shift test was grade 3. MRI revealed a rupture of the ACL on the femoral side and a bone bruise on the lateral femoral condyle and lateral tibial plateau (Fig. 2a and b). The patient underwent five PRP treatments at 1-week intervals from 4 weeks postinjury and resumed low-speed exercises from 6 weeks. At 10 weeks postinjury, the pivot shift test improved to grade 1, and the patient returned to all exercises. At 12 weeks, the KOOS was 84.3 (P: 90.0, S: 82.1, A: 89.7, SP: 75.0, Q: 62.5), and the patient was allowed to return to the match. Consequently, he competed 14 weeks postinjury, and his Tegner score recovered to 9, the preinjury level (Fig. 3b). At 4-month follow-up, the ACL was again clearly visualized on MRI (Fig. 2c), and the pivot shift test was grade 1. At the final 1-year follow-up, the patient had continued to compete without reinjury or compromising.

## 5 Discussion

This report presents cases of two high-impact sports athletes with ACL injuries who returned to the same preinjury level as early as 4 and 3.5 months, respectively, and continued to compete without losing stability at the final follow-up. Moreover, their KOOS exceeded the median score for athletes after ACL reconstruction, suggesting satisfactory performance. Although 90.5 % of patients treated conservatively resumed sports 13.8 weeks after an ACL injury, conscious performance was only 58.4 %, indicating that it is hard to combine early return with high performance [13]. In this context, some reports are available on the application of autologous platelet biotherapy, such as PRP, to treat ACL injury. PRP is considered to promote the healing of ligamentous tissues through the action of anti-inflammatory cytokines in platelets, the activation of mesenchymal cells and fibroblasts by various growth factors, such as platelet-derived growth factors, transforming growth factor (TGF)-β, vascular endothelial growth factor, and insulin-like growth factor (IGF-1), and the extracellular matrix synthesis [8], [14], [15]. In animal studies, PRP has been reported to promote the repair of damaged ACL [7]. In the athletes' treatment, the administration of PRP to high-level professional football players with partial ACL tears has been reported to return 81 % of players to their preinjury level in an average of 16 weeks [16]. In addition, PRP has been used in ACL reconstruction surgery, even though reconstructed grafts have been demonstrated to decrease the time to graft maturation by 48% [17] and reduce the incidence of retear by a third [18]. Perhaps, the growth factors in PRP promote graft maturation and alter the natural history of remodeling, resulting in earlier and firmer graft maturation, improving both the duration and quality of the healing process [19], [20]. In our cases, high-impact sports athletes returned to the same preinjury level, suggesting that PRP treatment could be an option for aggressive conservative treatment of ACL injuries. The application of PRP treatment to the treatment of ACL injuries may in the future increase the number of cases where surgery can be avoided, depending on the form of the tear and the length of time since injury. Nevertheless, as only two cases are available at present, further studies are warranted to compare our results with those of surgical treatment and conventional conservative treatment.

## 6 Conclusion

Two high-impact sports athletes with ACL injuries treated with aggressive conservative therapy using PRP returned early to their preinjury play level. Hence, PRP treatment could be utilized for aggressive conservative treatment of ACL injuries.

## Provenance and peer review

Not commissioned, externally peer-reviewed.

## Ethical approval

This report is a retrospective study, approved by the Ethics Committee (03-20) and has been reported in line with the SCARE criteria, and written informed consent was obtained from the patient for the use and publication of data for academic purposes. A copy of the written consent is available for review by the Editor-in-Chief of this journal on request.

## Funding

All authors declare that there is no funding regarding this case report.

## Guarantor

Shinnnosuke Hada MD, PhD.

## Research registration number

Not applicable.

## CRediT authorship contribution statement

Shinnnosuke Hada MD, PhD: study concept, data collection, data interpretation, and writing the paper
Muneaki Ishijima MD, PhD: Study concept and design
Hiroyuki Omiya PT; data collection
Yoshimasa Tomita MD: Study concept and design
Masao Hada MD, PhD; Study concept and design.

## Declaration of competing interest

All authors declare that there is no conflict of interest regarding this case report.
